# Multiple introductions of monkeypox virus to Ireland during the international mpox outbreak, May 2022 to October 2023

**DOI:** 10.2807/1560-7917.ES.2024.29.16.2300505

**Published:** 2024-04-18

**Authors:** Gabriel Gonzalez, Michael Carr, Tomás M Kelleher, Emer O’Byrne, Weronika Banka, Brian Keogan, Charlene Bennett, Geraldine Franzoni, Patrice Keane, Cliona Kenna, Luke W Meredith, Nicola Fletcher, Jose Maria Urtasun-Elizari, Jonathan Dean, Ciaran Browne, Fiona Lyons, Brendan Crowley, Derval Igoe, Eve Robinson, Greg Martin, Jeff Connell, Cillian F De Gascun, Daniel Hare

**Affiliations:** 1 UCD National Virus Reference Laboratory, University College Dublin, Dublin, Ireland; 2 Japan Initiative for World-leading Vaccine Research and Development Centers, Hokkaido University, Institute for Vaccine Research and Development, Sapporo, Japan; 3 International Collaboration Unit, International Institute for Zoonosis Control, Hokkaido University, Sapporo, Japan; 4 Department of Virology, St. James’s Hospital, Dublin, Ireland; 5 Department of Pathology, University of Cambridge, Cambridge, United Kingdom; 6 Veterinary Sciences Centre, University College Dublin, Dublin, Ireland; 7 Centre for Experimental Pathogen Host Research, University College Dublin, Dublin, Ireland; 8 National MPOX Crisis Management Lead, Acute Operations, Health Service Executive, Dublin, Ireland; 9 Sexual Health and Crisis Pregnancy Programme, Health and Wellbeing, Strategy and Research, Healthcare Strategy, Health Service Executive, Dublin, Ireland; 10 Health Service Executive Public Health: National Health Protection, Ireland; 11 Health Protection Surveillance Centre, Dublin, Ireland

**Keywords:** monkeypox, mpox, monkeypox virus, genome, epidemiological analysis, epidemic dynamics

## Abstract

**Background:**

Mpox, caused by monkeypox virus (MPXV), was considered a rare zoonotic disease before May 2022, when a global epidemic of cases in non-endemic countries led to the declaration of a Public Health Emergency of International Concern. Cases of mpox in Ireland, a country without previous mpox reports, could reflect extended local transmission or multiple epidemiological introductions.

**Aim:**

To elucidate the origins and molecular characteristics of MPXV circulating in Ireland between May 2022 and October 2023.

**Methods:**

Whole genome sequencing of MPXV from 75% of all Irish mpox cases (182/242) was performed and compared to sequences retrieved from public databases (n = 3,362). Bayesian approaches were used to infer divergence time between sequences from different subclades and evaluate putative importation events from other countries.

**Results:**

Of 242 detected mpox cases, 99% were males (median age: 35 years; range: 15–60). All 182 analysed genomes were assigned to Clade IIb and, presence of 12 distinguishable subclades suggests multiple introductions into Ireland. Estimation of time to divergence of subclades further supports the hypothesis for multiple importation events from numerous countries, indicative of extended and sustained international spread of mpox. Further analysis of sequences revealed that 92% of nucleotide mutations were from cytosine to thymine (or from guanine to adenine), leading to a high number of non-synonymous mutations across subclades; mutations associated with tecovirimat resistance were not observed.

**Conclusion:**

We provide insights into the international transmission dynamics supporting multiple introductions of MPXV into Ireland. Such information supported the implementation of evidence-informed public health control measures.

**Key public health message**

**What did you want to address in this study and why?**

We wanted to investigate the origins of monkeypox virus (MPXV) circulating in Ireland during the 2022–23 mpox outbreak to understand whether the virus was spread by local transmission or repeated importations. We also wanted to be able to detect genetic mutations in the virus associated with drug resistance.

**What have we learnt from this study?**

Multiple introductions of MPXV took place into Ireland during 2022–23 international outbreak, with limited evolution of the virus occurring locally. Mutations associated with drug resistance were not observed.

**What are the implications of your findings for public health?**

There is an ongoing risk of importations of MPXV into Ireland as the virus continues to circulate internationally. Such findings provide support for measures aimed at mitigating the spread of MPXV, such as targeted vaccination campaigns, self-isolation of mpox cases and community engagement, and emphasise the important role that pathogen genomics can play to support public health responses to emerging infectious diseases.

## Introduction

Monkeypox virus (MPXV) is a double-stranded DNA virus in the genus *Orthopoxvirus* within the *Poxviridae* family [1], causing the disease mpox. The MPXV genome is ca 197.2 kb encoding a predicted 191 proteins and is genetically closely related to the variola (smallpox) virus. Despite its name, the natural reservoir of the virus remains unknown, but it is transmitted among small mammals, such as rodents [2]. Two major clades of MPXV have been recognised as circulating endemically in Africa, one in Central Africa (Clade I) and the other one in West Africa (Clade II) with differing transmissibility and case fatality rates [3]. In May 2022, an increased number of mpox cases started appearing internationally [1,3], which were characterised as part of the West African Clade II. Based on viral phylogenetic properties and epidemiological evidence of increased human-to-human transmission outside of endemic geographic locations, MPXV causing the 2022 outbreak were classified within a distinct subclade, termed IIb [4]. The outbreak has been reported across multiple countries on different continents and differed from earlier outbreaks of Clade I and IIa in terms of patient age (54.3% of individuals in their 30s) and predominantly affecting men who have sex with men (MSM) [5].

Mpox clinical symptoms can last between 2 and 4 weeks and include fever, sore throat, headache, muscle aches, low energy, swollen lymph nodes and rash. However, the combination and severity of these symptoms varies among patients and some severe cases can develop skin lesions [2,6,7]. The overall case fatality rate of mpox has been estimated at 8.7%, though substantial differences have been observed between clades, estimated as 10.6% for Clade I vs 1–3.6% for Clade IIa [8,9]. The mortality rate in the range 0.01–1.81% for Clade IIb associated with the 2022–23 epidemic [10], and up to November 2023, the mortality rate has been 0.18% (171/92,783) [11]. However, cases could be underestimated because of resource-poor healthcare settings and asymptomatic cases [12]. 

The first detection of MPXV in Ireland was notified to the public health authorities on 31 May 2022, with the earliest onset of symptoms among confirmed cases on 13 May [13]. The peak of the outbreak nationally was observed in August 2022 and, while mpox cases have declined dramatically since this time in the context of a multi-faceted public health response, sporadic detections persisted in 2023. As such, investigating the patterns of introduction into the country, and the evolutionary characteristics of MPXV in Ireland is important to inform timely and effective public health measures, as the virus continues to circulate internationally. Clade case designation of MPXV by genomic analysis facilitates an evidence-based assessment of transmissibility and the ability to distinguish new importation events. Using whole genome sequencing (WGS) results from all available MPXV laboratory-confirmed positive cases, we aimed to ascertain whether the origin of mpox outbreak in Ireland was derived from a single introduction of the virus or from multiple importation events.

## Methods

### Sample and data collection

Samples comprising predominantly swabs of skin lesions/pustules transported in virus transport media (VTM) were received from 242 symptomatic individuals who presented for medical attention in Ireland. Samples were submitted to the National Virus Reference Laboratory (NVRL) and St James’s Hospital, Dublin, Ireland, between May 2022 and November 2023 for clinical diagnostic purposes by molecular methods, and for surveillance of mpox as a notifiable disease in Ireland (https://www.hpsc.ie/notifiablediseases/listofnotifiablediseases). 

Demographic data were extracted from publicly available national disease surveillance reports, produced by the Health Protection Surveillance Centre (HPSC, https://www.hpsc.ie/a-z/zoonotic/monkeypox/mpoxdataandreports).

### Sample extraction

Samples were processed following lysis and inactivation in a biosafety containment level 3 (BSL3) facility with a VTM to guanidinium isothiocyanate lysis buffer mixed in a ratio of 1:2.5. Total nucleic acids were extracted on the Roche MagNA Pure 96 platform as follows: 200 µL of swab material in VTM was added to 500 µL Roche lysis buffer, and, following external lysis, were extracted with an input volume 450 µL and elution in 100 µL. Alternatively, DNA from 200 µL of material in VTM was extracted using the Qiagen QIAamp DNA Mini kit, according to the manufacturer’s instructions.

### Molecular diagnostics

Real-time PCR for the detection of viral DNA was performed on the ABI7500 SDS platform (Applied Biosystems), employing initially the pan-Orthopoxvirus zoonotic (non-variola) RT-PCR kit (Altona) between May and July 2022. For logistical reasons, the assay was switched to the MPXV generic laboratory developed test based upon Li et al. [14] in August 2022, and subsequently to the LDT pan-Orthopoxvirus assay, in use since October 2022 [15], following a United States Centers for Disease Control and Prevention notification of a rare but significant deletion in the target TNF gene [16].

### Whole genome sequencing

This report considered only a single sample and corresponding genome sequence per clinical case. The MPXV qPCR positive samples were processed for WGS following a tiled amplicon and next-generation sequencing (NGS) approach employing an Oxford Nanopore Technologies (ONT) methodology. Tiled amplicons were generated from each sample employing two separate MPXV primer pools (P/N 50025, MBS), according to the manufacturer’s instructions [17]. Amplicons were barcoded employing the Rapid Barcoding Kit 96 (SQK-RBK110.96; ONT) and loaded onto a R9.4.1 flow cell (FLO-MIN106D; ONT) with 23 samples and a negative control on each run. The data generated were basecalled and demultiplexed with Guppy v5.1.13 (ONT) using the default parameters. The sequence reads were trimmed to remove adapters and barcode sequences with Porechop v.0.3.2pre (https://github.com/rrwick/Porechop). The NGS reads were mapped to an MPXV genome reference (GenBank ON676708) with Minimap 2.26 (https://github.com/lh3/minimap2) using the pipeline for Oxford Nanopore genome reads, i.e. ‘-ax map-ont’; mapped reads were manipulated with Samtools (https://www.htslib.org) to assess low support coverage and nucleotide sites with support coverage < 20 reads were assigned with ‘N’. The consensus sequences were obtained with seqtk (https://github.com/lh3/seqtk). The MPXV clades were assigned using the online tool in Nextclade for clade assignment (https://clades.nextstrain.org) with the database hMPXV updated on 1 August 2023 and with reference MPXV-M5312_HM12_Rivers (NC_063383.1). Also, Nextclade was used to infer nucleotide and amino acid substitutions relative to the reference genome. All multiple sequence alignments were performed with MAFFT by the algorithm FFT-NS-1 [18].

### Inferring time to the most recent common ancestor

The divergence times of the different MPXV subclades to the most recent common ancestor (tMRCA) were inferred using BEAST2 v2.6.7 [19]. The MPXV reference sequence ON676708 (Clade IIb A.1.1) was employed as an outgroup for rooting the tree. The substitution model was chosen using the model test in RAxML GUI [20] and the Bayesian information criterion (BIC), choosing the Hasegawa-Kishino-Yano (HKY) substitution model as the best fit for the sequences requiring the fewest number of parameters with a proportion of invariant sites (+ I). The different models with a constant clock or an uncorrelated optimised relaxed clock (ORC) [21], with the population modelled either as constant size, exponential growth or Bayesian skyline, were considered and compared to identify the most likely model among the six combinations. All models were calibrated with the collection date of the samples. Each model was executed for 1.5 x 10^8^, steps sampling every 10^4^ to assure an effective sample size (ESS) > 100 for all parameters in the models, i.e. the number of effectively independent draws for each parameter from the posterior distribution equivalent to the Markov chain. The model parameters were summarised and explored with Tracer 1.7.1 and the phylogenetic tree was inferred with TreeAnnotator in the BEAST bundle, removing 10% of trees of the chosen model as burn-in and using the common ancestor heights. Tree edition was performed with FigTree v1.4.4 (http://tree.bio.ed.ac.uk).

### Testing association with international monkeypox virus genome sequences

Contemporaneous sequences were downloaded from the MPXV database in GISAID (https://gisaid.org) [22] with the filtering criteria requiring near complete genome sequences and high quality (> 90% genome coverage). The accession numbers and metadata for the 3,362 GISAID records are provided in Supplementary Table S1. Additionally, reference sequences from previous MPXV outbreaks were downloaded from GenBank (Supplementary Table S1). The clade of these sequences was also inferred using Nextclade. The association of genome sequences from Ireland to sequences from other countries was tested by separating the group of sequences from Ireland into the following twelve IIb subclades: B.1, B.1.1, B.1.2, B.1.3, B.1.5, B.1.7, B.1.8, B.1.9, B.1.10, B.1.11, B.1.12 and C.1. The multiple sequence alignments of these groups included GISAID sequences from the same subclades to test the phylogenetic relationships and putative origins of the MPXV with the Bayesian inference approach of Outbreaker2 [23], using the collection date reported in GISAID and assuming an infectious period of 28 days with the highest probability of transmission between days 6 and 15, following the description of mpox cases and transmission via skin lesions [2]. The assumed mutation rate was 5 x 10^−5^, which was the lower end of the mutational rate range inferred from the BEAST analysis. The Outbreaker2 inference was run for 10^6^ steps in each of the 12 groups, with 10% burn-in and sampling every 2,500 steps. Among the Outbreaker2 results of transmissions, we considered as evidence of transmission between two samples those links with support > 0.1 and suggesting less than two generations of onward transmission.

## Results

### Characteristics of the mpox outbreak

**Figure 1:**
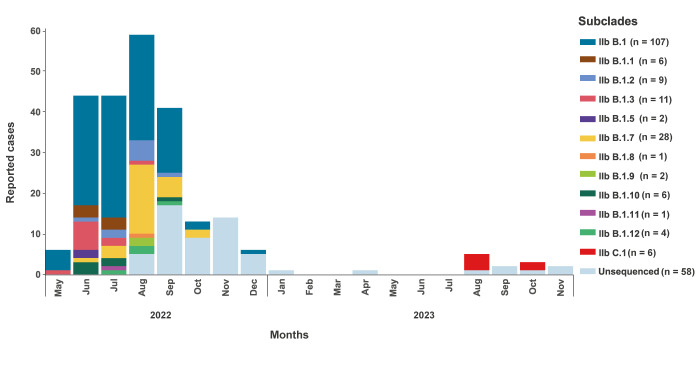
Reported mpox cases and monkeypox virus subclade, Ireland, May 2022–November 2023 (n = 241^a^)

^a^ One of the unsequenced samples lacked a collection date and is unaccounted for in the figure.

The 242 MPXV qPCR-positive samples analysed in the present study were collected between May 2022 and November 2023, representing all detected cases in Ireland. As reported by the HPSC of Ireland, 240 of these 242 mpox cases were males (99%) with a median age of 35 years (range: 15–60), with 79% (n = 189) aged between 18 and 44 years [13]; two cases were females. These cases were distributed over time with a peak in August 2022 (n = 59 cases) and the subclades were determined for those cases that were sequenced (Figure 1). 

Subclades assigned by Nextclade to sequences are coloured, and cases without a corresponding sequence are in grey. The total number of sequences per subclade is shown in parentheses.

### Whole genome sequencing of monkeypox virus

**Figure 2:**
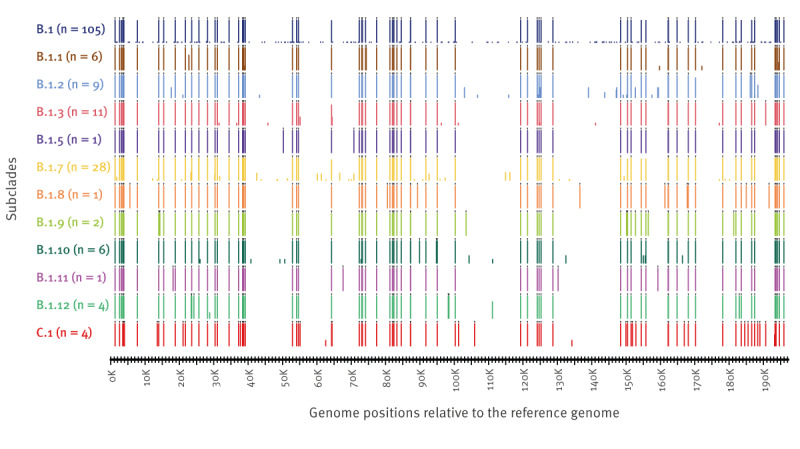
Distribution of mutations across monkeypox virus subclades, Ireland, May 2022–November 2023 (n = 373)

Of the 242 mpox cases, samples from 182 (75%) were successfully sequenced, of which 180 met the ≥ 90% coverage threshold with an average genome coverage of 99.5 ± 1.1%. The accession numbers and classification of the 182 sequenced genomes are provided in Supplementary Table S2. The high genome sequence coverage negatively correlated with the real-time PCR quantification cycle (Cq) values of the samples, with a Pearson’s correlation index r^2^ = 0.29 (p value = 4 x 10^−15^) and the Cq values in the 95% confidence interval (22.14–23.13). The samples with the lowest sequence coverage (< 95% genome coverage) were reported with Cq values > 30. The high coverage of the sequences facilitated the mutational analysis compared with the MPXV Clade IIb reference genome NC_063383.1. The transition/transversion ratio was 18.63, with 373 positions showing mutations relative to the reference genome. This ratio was considered as excessive compared with e.g. the transition/transversion ratio of 2.53 previously determined for cytomegalovirus, which is another linear double-stranded DNA virus with a genome size > 200 kb [24]. Of the total number of mutations, 45.0% (168/373) and 46.6% (174/373) were the transitions cytosine to thymine (C-to-T) and guanine to adenine (G-to-A), respectively (the summary of the mutations between different nucleotides is shown in Supplementary Table S3). Such transitions were more frequent in a 5’-TC-3’ context with 93.5% (157/168) of C-to-T mutations and 91.9% (160/174) of G-to-A, indicative of the effects of host apolipoprotein B mRNA editing catalytic polypeptide-like (APOBEC) cytidine deaminase enzymes [25]. Relative to the reference genome annotation, these mutations were approximately six times (322 vs 51) more frequent in coding regions than in non-coding regions (Supplementary Table S4). Moreover, 191 mutations were non-synonymous mutations while 131 were synonymous mutations and 51 characterised as non-coding mutations (Supplementary Table S4). These mutations were distributed into the different Nextclade-assigned subclades (Figure 2). A total of 57 mutations were common to all sequenced samples; 211 mutations were identified as isolated single nucleotide variants (SNV) present in only one sequence, while the remaining 105 mutations appeared in multiple, but not all, sequenced genomes (Supplementary Table S6 shows the detailed counts of sites with mutations per subclade). Of the 211 SNV occurring only once, 149 SNV were in the IIb B.1 subclade (dark blue sequences in Figure 2). Conversely, a total of 43 SNVs were distributed, characteristic to specific lineages, named as follows – B.1.1: 1 SNV (n = 6 sequences/subclade), B.1.2: 1 SNV (n = 9), B.1.3: 0 SNV (n = 11), B.1.5: 2 SNV (n = 1), B.1.7: 1 SNV (n = 28), B.1.8: 8 SNV (n = 1), B.1.9: 6 SNV (n = 2), B.1.10: 2 SNV (n = 6), B.1.11: 4 SNV (n = 1), B.1.12: 5 SNV (n = 4), and C.1: 13 SNV (n = 4) (Figure 2). The remaining 62 of the 373 mutations corresponded to variants within each subclade. These SNV have accumulated following divergence among subclades and point mutations were still accumulating, as was most evident in the basal subclade IIb B.1.

The horizontal axis corresponds to genome positions relative to the reference genome (NC_063383.1). The vertical axis corresponds to subclades identified in Ireland with the number of sequenced samples indicated in parentheses and are coloured by subclades as in Figure 1. Vertical lines mark the location of the mutations with the length of the line in proportion to the percentage of sequences in the subclade presenting the mutation. Mutations present in all sequences in the subclade are topped with a black triangle.

Among the predicted amino acids substitutions (details about mutations per gene are provided in Supplementary Table S4), only the substitution E353K was characterised in the OPG057 gene, which is an orthopoxviral F13L gene homologue and target of the antiviral drug tecovirimat [26]. Although this substitution is common to Clade IIb, it is not associated with tecovirimat resistance. In contrast, three amino acid substitutions, L108F, S432F and D740N, were found in OPG071, which encodes the DNA-directed DNA polymerase that is a target of the antiviral drugs cidofovir and brincidofovir [27].

**Figure 3:**
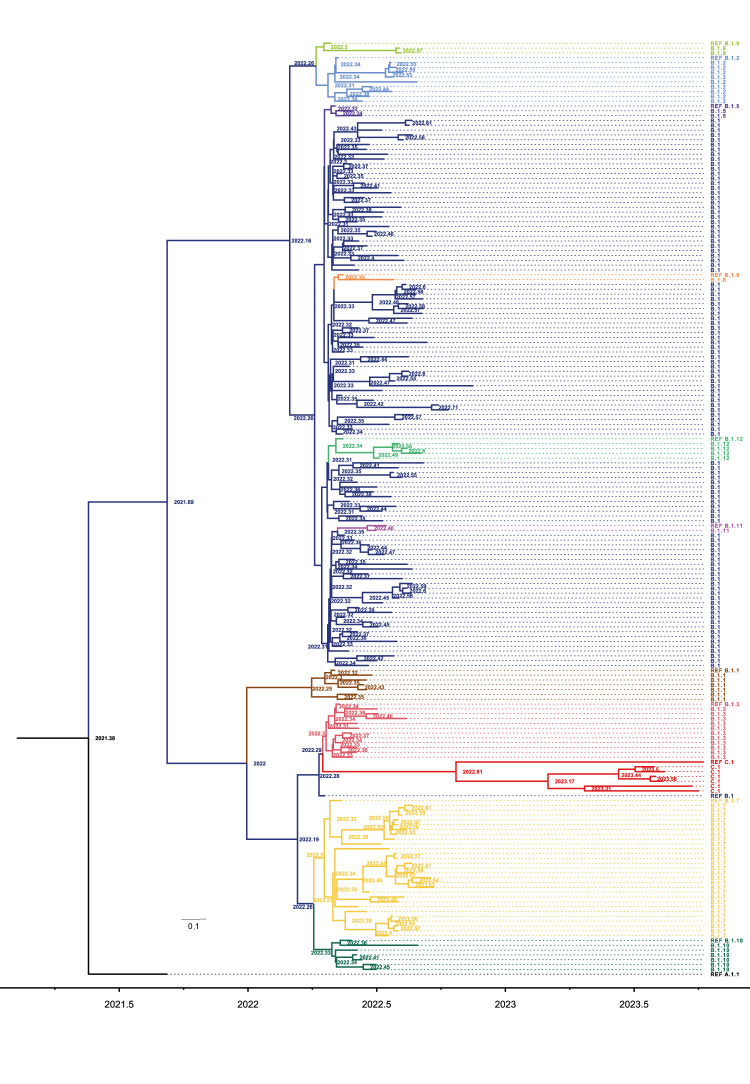
Bayesian-inferred phylogenetic tree with the most likely time of the most recent common ancestor for the monkeypox virus subclades, Ireland, May 2022–November 2023 (n = 194)

The subclades assigned by Nextclade, the number of characteristic mutations per subclade, and the phylogenetic relationships among our samples and sequences from previous outbreaks confirmed the identity of the sequences as belonging to the MPXV Clade IIb B.1 (Figure 3). Furthermore, at least 11 phylogenetically separable and highly supported subclades were suggested to circulate simultaneously in Ireland during the 2022 epidemic and thereafter a separate subclade in 2023 (Figure 3). The cases identified during the outbreak in 2022 belonged to the subclade IIb B.1. However, during the May 2022–November 2023 period when these cases were detected, distinct variants were identified, suggesting at least two alternative hypotheses to explain these findings: (i) subclades were introduced to Ireland in a single initial wave that evolved locally or (ii) a flow of new subclades to the country occurred in multiple separate importation events.

The phylogenetic tree represents the 181 Irish monkeypox virus genome sequences rooted with ON676708 (IIb A.1) and other reference genomes for the 12 subclades, indicated with REF in the tip names. The nodes show the most likely time between 2022 and 2023 for the most recent common ancestor. Subclades are coloured as in Figure 1 and are shown as tip names for clarity. The scale is shown in the figure representing approximately 0.1 nucleotide substitutions per site.

### Dating the divergence of monkeypox virus subclades

To further investigate the possible origins of the multiple subclades circulating in Ireland, the divergence times of the different subclades were inferred employing a Bayesian approach. The comparison of the considered Bayesian models demonstrated that an uncorrelated relaxed clock was the model with the highest likelihood for the observed genomic data (Supplementary Table 5), while the coalescent population models shared similar likelihoods, with the Bayesian skyline reflecting better the increment of cases experienced in 2022 and a relative reduction in 2023. The dating of the tMRCA of the different subclades to October 2021 (2021.73: 95% highest posterior density (HPD) (2021.16–2022.12)) was consistent with divergence before the outbreak in Ireland (Supplementary Table 5, Figure 3). Furthermore, while other phylogenetic groups were observed to have diverged as early as the last week of May, the samples from Ireland of at least three clades, namely IIb B.1.9, IIb B.1.11 and B.1.12, diverged in July and August 2022. Therefore, given that no earlier cases assigned to these clades were detected, it is considered more likely they were introduced into the country later in the course of the outbreak. Additionally, other sub-clusters in IIb B.1, B.1.2 and B.1.7 are suggested to have diverged after August and as late as September 2022 (Figure 3). It is noteworthy that despite the best fit being a Bayesian skyline with the ORC, all the inferred models had very similar dates for the tMRCA and relatively high mutation rates in the range of 5.9×10^−5^–1.4×10^−4^, which is notably higher than the 10^−6^ mutation rate previously reported for the genus *Orthopoxviruses* [28].

### Uncovering possible origins of the monkeypox virus introductions to Ireland

**Figure 4:**
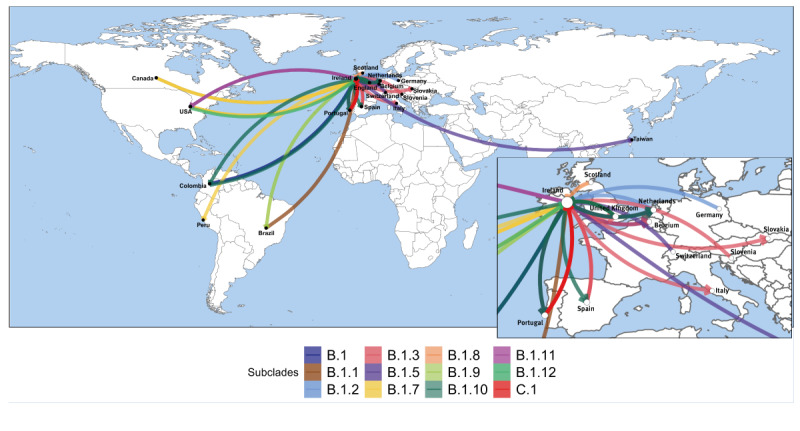
Summary of inferred monkeypox virus international transmissions among countries with genetically closely related subclades detected in Ireland, May 2022–November 2023

The Clade IIb subclade B.1 was predominant during the 2022–23 MPXV outbreak in Ireland, identified in 59% (n = 107) of the cases, and consistent with 66% (670/976), 45% (528/932) and 37% (289/487) of GISAID-deposited sequences from Europe, North America and South America from this subclade, respectively (Table). As some of the other circulating subclades were suggested to have diverged in the month before the first mpox case was detected in Ireland (31 May 2022), the subclade sequences were compared with sequences from other geographical locations to identify putative origins for the different clades and to further explore the evidence for any transmission events among these mpox cases (Figure 4). Despite the conservative approach used to infer the transmission, i.e. requiring less than two generations and a support of > 10% in the Outbreaker2 inference, sequences from many different countries were inferred to be connected to sequences from Ireland, reflecting the complex dynamics of international MPXV transmission in the 2022 global outbreak. The Bayesian analysis inferred strong support for importation events based on the collection dates and phylogenetic relationships among the sequences, including importations into Ireland from Europe (United Kingdom, Portugal, Germany, Switzerland), North America (United States, Canada) and South America (Colombia, Brazil) (Figure 4). Also, transmissions were inferred from Ireland to other countries such as Spain, Portugal, Slovakia, Italy and Belgium (Figure 4). Interestingly, although the subclade IIb C.1 has been observed to be most characteristic of samples collected in Asia (representing 49% (84/171)) (Table), the introduction to Ireland was inferred to be most likely related to cases sequenced in Portugal (Figure 4).

**Table:** Monkeypox virus Clade IIb subclades in GISAID, Ireland and worldwide, 2022–2023

Subclades	Location^a^												GISAID Sequences^b^	
	Ireland		Europe		North America		South America		Asia		Oceania			
	n	%	n	%	n	%	n	%	n	%	n	%	n	%
B.1	109	59	670	66	528	45	289	37	9	5	2	6	1,607	48
B.1.1	6	3	57	6	15	1	71	9	3	2	0	0	152	5
B.1.2	9	5	38	4	136	12	13	2	7	4	0	0	203	6
B.1.3	11	6	32	3	49	4	0	0	1	1	15	47	108	3
B.1.5	2	1	18	2	15	1	0	0	1	1	0	0	36	1
B.1.7	29	16	50	5	68	6	2	0	2	1	0	0	151	5
B.1.8	1	1	16	2	23	2	4	1	0	0	0	0	44	1
B.1.9	2	1	31	3	1	0	50	6	0	0	0	0	84	2
B.1.10	6	3	9	1	9	1	35	4	0	0	0	0	59	2
B.1.11	1	1	1	0	45	4	21	3	0	0	0	0	68	2
B.1.12	4	2	4	0	43	4	2	0	0	0	0	0	53	2
C.1	4	2	50	5	0	0	0	0	84	49	15	47	153	5
Others^c^	0	0	39	4	246	21	295	38	64	37	0	0	642	19
**Total sequences^d^**	**184**	**100**	**1,015**	**100**	**1,178**	**100**	**782**	**100**	**171**	**100**	**32**	**100**	**3,362**	**100**

^a^ Geographical location data summarised as reported in GISAID.

^b^ Total of sequences for the corresponding subclade.

^c^ Summary of other subclades not identified in Ireland yet.

^d^ Total sequences for the corresponding geographical location.

Different countries around the globe for which international transmissions were inferred. The lines represent inferred migratory events between countries where subclades in the Clade IIb B.1 were sequenced. The arrows represent the direction of inferred transmission events and the colours to the subclades as shown in the legend.

## Discussion

Our results from the MPXV genomic epidemiological analysis strongly supports multiple introductions of the virus into Ireland during 2022, with evidence for ongoing divergence during 2023. Such findings are consistent with national published reports documenting that 48% (116/242) of cases were associated with a recent history of international travel in the 21 days before symptom onset [13]. Our analyses also contextualised mpox cases detected in Ireland within the global genomic-epidemiological landscape of the international MPXV 2022 outbreak [29,30], with support for subclades present at higher prevalence in other geographic regions, as importation events into Ireland.

The major peak of mpox cases was in August 2022 and cases have declined dramatically since this time. A similar observation has been shared in other countries where the numbers of cases have also decreased significantly. The explanation for the decline in case numbers is likely multifactorial including effective public health communication regarding protective measures and the subsequent introduction of prevention practices, i.e. vaccination, self-isolation, to mitigate against transmission in vulnerable groups. In parallel, the introduction of a targeted vaccination policy to protect those at most risk and the reduced mass gathering events in 2022, because of the COVID-19 pandemic, likely also reduced further the number of mpox cases [31]. The third-generation smallpox vaccine (Modified Vaccinia Ankara – Bavarian Nordic (MVA-BN)) was offered as mpox pre- and post- exposure prophylaxis and it has been suggested that the previous cessation of the smallpox vaccination around the world could have been one of the factors contributing to the initial cases of the current outbreak [8].

The relatively small increase in the number of reported cases in Ireland in the second half of 2023, and internationally a surge of cases in East Asia during July and August [32], indicates importation events are likely occurring with the potential for onward transmission [33]. Such cases highlight the need for vigilance, vaccination and early detection with timely genomic surveillance and rapid public sharing of sequences to monitor for potential amino acid substitutions and for MPXV clade changes over time on both national and international levels [34]. The availability of the large genomic epidemiological baseline from 2022 to 2023 described in this study will facilitate the delineation of transmission patterns and, specifically, the ability to distinguish imported from autochthonous transmission events as the priority must be to mitigate against further disease transmission by preventing the establishment of recalcitrant reservoirs arising from potential silent transmission, i.e. in asymptomatic cases [12], in different geographical regions which may seed further outbreaks.

The dominant MPXV clade circulating in Ireland was suggested to accumulate mutations at a rate that was at least an order of magnitude higher than previously reported [28], which is consistent with recent data from a number of other studies [35,36]. The Bayesian-inferred population model suggested an increase in the effective population size early on in the outbreak that gave rise to many of the different subclades, as a probable consequence of the extensive human-to-human transmission events, and adaptation and modification by the human host immune response [31]. Such results could explain the distinctive pattern of the number of accumulated mutations as the virus was replicating and being transmitted. The analysis of mutational patterns among our MPXV cases strongly supported the hypothesis that much of this divergence is driven by the effects of enzymes of the APOBEC cytosine deaminase family into dinucleotides where CT-to-TT mutations (or GA-to-AA on the lagging strand) predominated [29], as was also observed in the evolution of SARS-CoV-2 during the COVID-19 pandemic [37]. Therefore, while MPXV may still be considered a zoonotic infection [2], viral phylogenetic and Bayesian analysis of local and international viral genomes provides evidence to support sustained adaptation of the virus to the human host, posing ongoing risk to public health by altered viral transmission, in particular close physical contact and the continued need for vigilance [31,36].

The notably high sequencing coverage in our study facilitated robust phylogenetic inferences to be drawn by reducing uncertainty as to the direction and dating of the transmission between cases. With a lower level of national genomic surveillance, uncertainty increases and the number of possible transmission routes and epidemiological variables, e.g. prevalence in the country and possible relation among cases, also grow exponentially. As a consequence, this limits the amount of information that can be drawn from the gathered data. The combination of epidemiological data with high sequencing coverage for a particular outbreak can lead to insights into the origins, the transmission dynamics and even susceptibility of different populations, as was illustrated during the COVID-19 pandemic in tracing the epidemiological dynamics of SARS-CoV-2 [38,39].

Although this report presents a high percentage of sequenced cases, a number of limitations to the data should be acknowledged. Firstly, as the genome coverage of samples is a function of the viral load in the collected samples, weak positive samples (Cq values > 30) were not determined, consequently limiting the transmission network inference. Secondly, the capacity to completely resolve the transmission networks by WGS may be limited by the possibility of asymptomatic mpox transmission [12]. Thirdly, differences in percentages of sequenced cases between countries impact the links that can be uncovered by the transmission analysis. Finally, despite the high mutation rate characterised for MPXV, inferring the direction of the international transmissions relied on the phylogenetic relationship among very similar sequences and the availability of accurate collection dates limiting the support for the inferred direction of the transmission events to the accuracy of the sequences and dates reported by different countries.

## Conclusions

In conclusion, this detailed viral phylogenetic analysis of mpox cases detected in Ireland during the 2022‒23 international outbreak provides evidence for multiple introductions of the virus into Ireland, and underscores the potential for future importation events. In the context of high sequencing coverage by international standards, our dataset provides an important genomic-epidemiological baseline to monitor future MPXV importation events. Our findings also highlight the need for ongoing vigilance as MPXV continues to circulate internationally, and contribute to the evidence base for the planning and implementation of future public health measures. Surveillance of viral genetic diversity can also facilitate the monitoring of genetic determinants associated with antiviral resistance, and the rational development of target-specific vaccines.

## Supplementary Data

358000 Supplement
