# Supplementary material for: Multiple introductions of monkeypox virus to Ireland during the international mpox outbreak, May 2022 to October 2023
Source: Euro Surveill. 2024 Apr 18;29(16):2300505. doi: 10.2807/1560-7917.ES.2024.29.16.2300505 (PMC11027473; doi:10.2807/1560-7917.ES.2024.29.16.2300505)
Supplement: Supplement [file 23-00505_GONZALEZ_Supplement.pdf]

Supplementary Materials

This supplementary material is hosted by Eurosurveillance as supporting information alongside the article [**Multiple introductions of monkeypox virus to Ireland during the international mpox outbreak, May 2022 to October 2023**] by Gabriel Gonzalez *et al.*, on behalf of the authors, who remain responsible for the accuracy and appropriateness of the content. The same standards for ethics, copyright, attributions and permissions as for the article apply. Supplements are not edited by Eurosurveillance and the journal is not responsible for the maintenance of any links or email addresses provided therein.

Supplementary Table S1. Reference sequences from GISAID and GenBank considered for the analyses.

Hosted in Zenodo.org as https://doi.org/10.5281/zenodo.10531878 (*Supplementary Table 1.xlsx*)

Supplementary Table S2. Samples sequenced in the Republic of Ireland during the 2022-2023 MPXV epidemic.

Hosted in Zenodo.org as https://doi.org/10.5281/zenodo.10531878 (*Supplementary Table 2.xlsx*)

Supplementary Table S3. Count of positions with mutations across the MPXV whole-genome sequences

		TO			
		**A**	**T**	**G**	**C**
**FROM**	**A**	-	2	7	2
	**T**	6	-	0	5
	**G**	**174**	5	-	2
	**C**	2	**168**	0	-

Supplementary Table S4. Distribution of mutations per coding regions.

Hosted in Zenodo.org as https://doi.org/10.5281/zenodo.10531878 (*Supplementary Table 4.xlsx*)

Supplementary Table S5. Comparison of Bayesian models and the inferred time to the MRCA of the MPXV clades in Ireland.

		Bayesian Model to Estimate time of the MRCA						
	**Population**	Constant Size	Constant Size	Exponential Growth	Exponential Growth	Bayesian skyline	**Bayesian skyline**	**Week (Month)**
	**Molecular clock**	Strict Clock	ORC	Strict Clock	ORC	Strict Clock	**ORC**	
**Mutation Rate**		7.95E-05	1.00E-04	7.55E-05	9.78E-05	7.27E-05	**7.64E-05**	
**95% HPDI**		[6.5E-05,9.6E-05]	[6.8E-05, 1.5E-04]	[6.0E-05, 9.13-05]	[6.4E-05, 1.4E-4]	[5.9E-5, 8.7E-5]	**[5.6E-5, 1.0E-4]**	
**Likelihood**		-267348	-267280	-267346	-267280	-267342	**-267280**	
**Date of MRCA**<br>**95% HPDI**	IIb B.1	2021.19<br>[2020.77, 2021.55]	2021.06<br>[2016.88, 2022.06]	2021.21<br>[2020.81, 2021.57]	2021.48<br>[2020.76, 2021.95]	2021.21<br>[2020.79, 2021.58]	**2021.73**<br>**[2021.16, 2022.12]**	2021 W40<br>(26 Sep-2 Oct)
	IIb B.1.1	2022.27<br>[2022.10, 2022.37]	2022.18<br>[2021.78, 2022.36]	2022.27<br>[2022.10, 2022.37]	2022.2<br>[2021.86, 2022.36]	2022.31<br>[2022.17, 2022.37]	**2022.29**<br>**[2022.09, 2022.37]**	2022 W22<br>(10-16 Apr)
	IIb B.1.2	2022.33<br>[2022.24, 2022.39]	2022.33<br>[2022.16, 2022.39]	2022.33<br>[2022.23, 2022.39]	2022.33<br>[2022.17, 2022.39]	2022.36<br>[2022.29, 2022.39]	**2022.35**<br>**[2022.30, 2022.39]**	2022 W19<br>(1-7 May)
	IIb B.1.3	2022.33<br>[2022.25, 2022.38]	2022.31<br>[2022.14, 2022.39]	2022.33<br>[2022.23, 2022.39]	2022.31<br>[2022.16, 2022.39]	2022.35<br>[2022.30, 2022.39]	**2022.35**<br>**[2022.29, 2022.39]**	2022 W19<br>(1-7 May)
	IIb B.1.5	2022.24<br>[2022.02, 2022.38]	2022.34<br>[2022.20, 2022.38]	2022.22<br>[2021.98, 2022.38]	2022.35<br>[2022.218, 2022.39]	2022.34<br>[2022.16, 2022.39]	**2022.37**<br>**[2022.32, 2022.39]**	2022 W20<br>(8-14 May)
	IIb B.1.7	2022.2<br>[2022.02, 2022.33]	2022.31<br>[2022.07, 2022.40]	2022.18<br>[2022.0, 2022.33]	2022.3<br>[2022.08, 2022.4]	2022.29<br>[2022.15, 2022.38]	**2022.34**<br>**[2022.25, 2022.39]**	2022 W19<br>(1-7 May)
	IIb B.1.8	2022.4<br>[2022.33, 2022.41]	2022.4<br>[2022.30, 2022.41]	2022.38<br>[2022.32, 2022.41]	2022.4<br>[2022.30, 2022.41]	2022.39<br>[2022.35, 2022.41]	**2022.39**<br>**[2022.35, 2022.41]**	2022 W21<br>(15-21 May)
	IIb B.1.9	2022.28<br>[2022.15, 2022.36]	2022.3<br>[2022.07, 2022.37]	2022.28<br>[2022.15, 2022.36]	2022.3<br>[2022.11, 2022.36]	2022.32<br>[2022.23, 2022.36]	**2022.34**<br>**[2022.27, 2022.37]**	2022 W19<br>(1-7 May)
	IIb B.1.10	2022.39<br>[2022.32, 2022.44]	2022.4<br>[2022.29, 2022.45]	2022.39<br>[2022.30, 2022.44]	2022.4<br>[2022.28, 2022.45]	2022.38<br>[2022.32, 2022.42]	**2022.37**<br>**[2022.30, 2022.41]**	2022 W20<br>(8-14 May)
	IIb B.1.11	2022.52<br>[2022.47, 2022.54]	2022.53<br>[2022.48, 2022.54]	2022.52<br>[2022.46, 2022.54]	2022.53<br>[2022.47, 2022.54]	2022.51<br>[2022.45, 2022.54]	**2022.51**<br>**[2022.43, 2022.54]**	2022 W28<br>(3-9 Jul)
	IIb B.1.12	2022.35<br>[2022.24, 2022.41]	2022.37<br>[2022.19, 2022.41]	2022.35<br>[2022.23, 2022.41]	2022.37<br>[2022.23, 2022.41]	2022.38<br>[2022.32, 2022.41]	**2022.39**<br>**[2022.34, 2022.41]**	2022 W21<br>(15-21 May)
	IIb C.1	2023<br>[2022.75, 2023.20]	2022.9<br>[2022.65, 2023.21]	2022.93<br>[2022.68, 2023.16]	2022.8<br>[2022.57, 2023.14]	2022.93<br>[2022.68, 2023.16]	**2022.85**<br>**[2022.61, 2023.17]**	2022 W47<br>(13-19 Nov)

Supplementary Table S6. Distribution of mutations across subclades identified in Ireland (n = 373).

		Number of Mutations[1]				
**Subclade**	**Sequences**	**In all subclade sequences[2]**	**Single sequence**	**Characteristic to subclade**	**Common to multiple subclades[3]**	**Total**
**B.1**	105	59	149	0		
**B.1.1**	6	68	4	1		
**B.1.2**	9	67	9	1		
**B.1.3**	11	67	7	0		
**B.1.5**	1	69	2	2		
**B.1.7**	28	64	19	1		
**B.1.8**	1	75	8	8		
**B.1.9**	2	73	0	6		
**B.1.10**	6	69	6	2		
**B.1.11**	1	71	4	4		
**B.1.12**	4	73	1	5		
**C.1**	4	82	2	13		
**Total**	***178***	***57***	***211***	***43***	***62***	***373***

[1] Sites with mutations relative to the reference MPXV-M5312_HM12_Rivers (NC_063383.1).

[2] Count of mutations present in all sequences of the respective subclade. The count for the total corresponds to the number of mutations present in all sequences.

[3] Sites with mutations present in multiple detected subclades but not in all subclades or sequences.
